# Reduction of the Bitter Taste in Packaged Natural Black Manzanilla Olives by Zinc Chloride

**DOI:** 10.3389/fnut.2018.00102

**Published:** 2018-10-26

**Authors:** Joaquin Bautista-Gallego, Francisco Rodríguez-Gómez, Verónica Romero-Gil, Antonio Benítez-Cabello, Francisco N. Arroyo-López, Antonio Garrido-Fernández

**Affiliations:** Food Biotechnology Department, Agencia Estatal Consejo Superior de Investigaciones Científicas, Instituto de la Grasa, Campus Universitario Pablo de Olavide, Seville, Spain

**Keywords:** preservation, natural black table olives, packaging, zinc fortified olives, sensory analysis

## Abstract

The work assays the use of various concentrations of ZnCl_2_ (0.0–0.1%, w/v) in packaged natural black Manzanilla table olives. The transformations were followed for 4 months. The presence of Zn modified the leaching of total sugars (sucrose, glucose, fructose, and mannitol) into the brine, which decreased as the ZnCl_2_ content increased. Over the study, sucrose and glucose were exhausted while fructose, although consumed, left some final residues and the use of mannitol was limited. Titratable acidity was always gradually formed causing the subsequent pH decrease, which stabilized at ≈3.5. Acetic and mainly lactic acid were also formed during the assay, reaching the highest level of lactic acid in the 0.050% ZnCl_2_ treatment, followed by the Control. The acids were formed by lactic acid bacteria (LAB) (*Lactobacillus pentosus*, 39%, and *Lactobacillus plantarum*, 61%). However, the most outstanding Zn effect was found on the olive sensory characteristics: its presence markedly reduced the bitter notes, increased the overall appreciation, and the treatment containing 0.075% ZnCl_2_ had the highest scores in hardness, crunchiness, and overall appreciation. Therefore, the addition of ZnCl_2_ into packaged natural table olives may lead to healthy products with desirable sensory characteristics which, in turn, could promote consumption.

## Introduction

The world consolidated table olive production for 2014/15 season was 2.6 × 10^6^ tons with the main producers being the EU (0.9 × 10^6^), followed by Egypt (0.5 × 10^6^), Turkey (0.4 × 10^6^), and Algeria (0.2 × 10^6^) (1). Unfortunately, the International Olive Council does not provide information on the distribution among types; however, it is well known that countries like Egypt, Turkey, Algeria, or Greece are great producers of natural black table olives. The most worldwide famous natural black olive presentations are based on the Greek Kalamata variety (2). In general, table olives are a good source of polyphenols, but hydroxytyrosol, tyrosol, tyrosol acetate or 1-acetoxy pinoresinol (3) and triterpenic acids (mainly maslinic and oleanolic acids, with ≈ 2000 mg/kg flesh), among others, are in higher concentrations in natural olives than in Spanish-style in which the lye treatment and washing cause partial leaching (4, 5). The high concentrations of triterpenes give natural olives antitumoral, cardioprotective, anti-inflammatory, and antioxidant activities (6). Also, natural black olives are an excellent source of anthocyanins, responsible for their pink-purple color (3, 7). The particular nutritional characteristics of natural olives are favored by the soft processing, which only implies a direct brining followed by spontaneous fermentation. The process produces then, a markedly reduced volume of wastewaters concerning other table olive methods. Natural black olives are usually packaged in brine, frequently added with various seasoning materials. Among this presentation, the Greek Kalamata olives, which typically include vinegar and good quality olive oil in the initial cover brine, are particularly appreciated by consumers. However, the packaged natural black table olives require for its stabilization pasteurization or preservatives, which may deteriorate the sensory characteristics (8).

Zinc chloride (ZnCl_2_) has shown in the synthetic medium good inhibitory effect against yeasts strains of the genera *Saccharomyces, Wickerhamomyces, Debaryomyces, Issatchenkia, Candida, Pichia, Kluyveromyces*, and *Torulaspora* isolated from table olives (9). This effect could open new alternatives to the use of this salt in fermented vegetables, which are usually prone to spoilage by yeasts, not only as a fortifying agent but also as a possible preservative (9). Its application at 0.050 and 0.075% ZnCl_2_ levels in cracked Manzanilla-Aloreña table olive packaging led to a significant reduction in the *Enterobacteriaceae* and yeast populations and increased the lactic acid bacteria (LAB) population (10). Also, the *Aloreña de Málaga* prepared with 0.075% ZnCl_2_ in the cover brine represented a novel Zn fortified table olive presentation (11). On the contrary, ZnCl_2_ added to green Spanish-style table olive packaging showed a lower inhibitory effect than potassium sorbate against yeasts (12). The loss of inhibitory efficiency could not be attributed to hydroxytyrosol or NaCl since, in their presence, the fungicidal activity of ZnCl_2_ and ZnSO_4_ against table olive yeasts, using synthetic medium, remained inalterable (13). Therefore, the role of zinc salts in table olives still requires further investigation even more in natural black table olives where there are no studies about zinc use. Also, the zinc is an essential trace element for humans due to its role in many physiological functions in the living systems (14). Diverse zinc salts can be used in the manufacture of food supplements (15). Therefore, the investigation of Zn effects on table olive products and its possible use as a fortifying agent or preservative is of scientific and practical interest.

The objective of this work was to study the transformations on the physicochemical, microbiological, and sensory characteristics of natural black Manzanilla table olives caused by the presence of ZnCl_2_ in the packaging brine. The addition may also enhance the healthy properties of the natural olive compounds with those provided by the zinc. Then, the product could represent an attractive, functional table olive trade preparation.

## Materials and methods

### Experimental design and samples

The natural black stored in brine Manzanilla olives used in the present study were provided by a local producer of the region of Seville (Spain). The pitted stored fruits were placed in plastic bags (420 g olives + 350 ml brine) and seasoned, as usual, with small pieces of thyme. Then, four different brines were used for packaging in bags. The first (Control) consisted of the brine habitually used by the industry, which contained (w/v): 0.4% potassium sorbate, 0.6% benzoic acid, 2.0% NaCl, 0.2% citric acid, 0.6% of ascorbic acid, and 0.25% of lactic acid. The other three brines were similar to that used by the producer, except for the absence of preservatives, but added with 0.050, 0.075, and 0.100% (w/v) ZnCl_2_, respectively. After filling, a total of 22 containers per treatment were closed and kept at room temperature (20 ± 3°C) for a period similar to that of real shelf life (4 months). Periodically, two replicate containers per treatment were removed and analyzed in duplicate. The first samples were collected 4 h after packaging.

### Physicochemical analysis

The analysis of brines for pH, chloride concentration, titratable acidity, and combined acidity were carried out using the standard methods developed for table olives (8). The concentrations of sugars and organic acids in brines were determined by HPLC according to the methods developed by Rodríguez-Gómez et al. (16).

CIE coordinates L^*^ (lightness), a^*^ (negative values indicate green while positive values indicate magenta), and b^*^ (negative values indicate blue and positive values indicate yellow) of the fruits were obtained using a BYK-Gardner Model 9000 Color-view spectrophotometer. Samples were covered with a box with a matt black interior to prevent interference by stray light. Each determination was the average of 20 olive measurements. The color index (Ci) was estimated using the formula:

(1) $$C_{i}=\frac{-2R_{560}+R_{590}+2R_{630}+2R_{640}}{3}$$

where the Rs stand for the reflectance values at 560, 590, 630, and 640 nm, respectively (17).

Instrumental firmness was measured using the protocol described by Bautista-Gallego et al. (18). Briefly, a Kramer shear compression cell coupled to an Instron Universal Testing Machine (Canton, MA) was used. The crosshead speed was 200 mm/min. The firmness (shear compression force) was the mean of 20 measurements, each of which was performed on one cracked pitted fruit from which the stone had been previously removed. The result was expressed as kN/100 g pitted olive.

### Microbiological analysis

Brine samples and their decimal dilutions were plated using a Spiral System model dwScientific (Dow Whitley Scientific Limited, England) on the appropriate media. Subsequently, plates were counted using a CounterMat v.3.10 (IUL, Barcelona, Spain) image analysis system, and the results expressed as log_10_ CFU/mL. *Enterobacteriaceae* were counted on VRBD (Crystal-violet Neutral-Red bile glucose)-agar (Merck, Darmstadt, Germany), total viable counts were determined on plate count agar (tryptone-glucose-yeast) (Oxoid), LAB on MRS (de Man, Rogosa and Sharpe)-agar (Oxoid) with 0.02% (w/v) sodium azide (Sigma, St. Luis, USA), and yeasts on YM (yeast-malt-peptone-glucose medium)-agar (DifcoTM, Becton and Dickinson Company, Sparks, MD, USA) supplemented with oxytetracycline and gentamicin sulfate as selective agents for yeasts. Plates were incubated in aerobiosis at 30°C for 48–72 h. Changes in the microbial populations were assessed by counts at selected sampling times.

Brine samples (100 mL) were collected in sterile conditions at the end of the study (~4 months) and plated on the yeast and LAB selective media described above. A total of 160 isolates, 80 LAB, and 80 yeasts (10 for each treatment and replicate) were randomly selected and purified by subsequent re-streaking on YM or MRS agar, respectively. The different LAB isolates were identified at species level using multiplex PCR analysis of the *rec*A with species-specific primers for *Lactobacillus pentosus, Lactobacillus plantarum*, and *Lactobacillus paraplantarum*, following the protocol described by Torriani et al. (19). Yeasts were stored at −80°C and subjected to further identification, probiotic, and technological tests which will be considered separately.

### Sensory analysis

The sensory analysis was conducted by a panel of 13 experienced judges (6 men and 7 women) from the staff of the Food Biotechnology Department. All panelists had participated in previous table olive classification tests (20, 21), had between 4 and 15 years' experience, and were familiar with sensory studies on table olive presentations. The tests were performed in individual booths under controlled conditions of light, temperature, and humidity. For the analysis, the descriptors included in the Sensory Analysis for Table Olives (22) sheet, added with an overall appreciation, were used, due to its familiarity to panelists. Despite this background, the panelists were trained for the proper assessment of the descriptors and the objectives of this experiment (23). The samples were served in the cups described in the Method for Sensory Analysis of Table Olives (24), coded with a 3-digit random number, and presented in a randomized balanced order to the panelists (25). Only four runs per session were performed. Samples were analyzed in triplicate.

The olives were scored according to a 10-cm unstructured scale. Anchor ratings were 1 (no perception) and 11 (extremely strong) for gustatory perceptions and low and high levels for kinesthetic sensations (26). The marks on the evaluation sheet were transformed into data by taking measurements (in 0.1 cm precision) from the left anchor.

Olives from the various treatments were also subjected to comparisons among them, using the sheets developed for the R-index. In these tests, panelists were asked to look for differences between a previously known sample and those supplied during the test (including the reference sample). The answers to be selected were S-sure (S), S-unsure (S?), N-unsure (N?), and N-Sure (N) and their preferences. Data collected were processed according to Lee and Van Hout (27).

### Statistical analysis

Data were processed using diverse statistical programmes according to the objective of the analysis, using ANOVA to study significant differences among treatments at particular sample points when necessary. Sigma Plot 13 (Systat Software, Inc.) was also used for most graphic presentations. Regarding the sensory analysis, the study of the panel behavior and characterization of products were performed using XLSTAT for Excel v 19.4 (AddisonSoft, France) and Panel Check v1.4.2 (a joint project by Nofima Mat., Technical University of Denmark, and the Department of Food Science of the University of Copenhagen). The data were subjected to ANOVA, considering the effects of treatments as fixed and those of assessors, sessions and their interactions as random, and other statistical tools provided by the packages. When appropriate, data were also auto-scaled and centered (28), to prevent bias due to differences in scales, or centered by assessors, a transformation that is particularly recommended when the analysis is focussed on the products and not on panelists, as in this study (29).

All treatments and attributes were also subjected to PCA, which is a procedure for condensing the maximum amount of variance in the minimum number of uncorrelated variables (usually called principal components or Factors) to prevent multicollinearity (30).

## Results and discussion

### Changes in the physicochemical characteristics of brines along the storage

#### Sugars

After 4 h packaging, there were remarkable initial concentrations of total sugar in brines which ranged from 0.95(±0.05) g/L, in the Control, to 0.61 (±0.01) g/L, in the 0.100% ZnCl_2_ treatments. The presence of sugars in the initial packaging brines are a consequence of their high levels in the raw materia used for the presentations which, in turn, is due to their slow diffusion from the plain fruits into the brines during the previous storage, caused by the limited permeability of the skin when olives are not lye treated (8). However, after pitting, the solubilisation was faster and the concentrations only a few hours after packaging reached considerable levels (Table 1). The differences between the Control (without Zn) and the treatments with the two highest concentrations of zinc salt used were significant at *p* < 0.05 but not with respect to those containing only 0.050% ZnCl_2_. Besides, there was no significant total sugar content between treatments with the two intermediate level of the salt (Table 1). In any case, the averages of the initial total sugar concentration as the Zn content increased was progressively lower. This delay in the sugars' release in the presence of Zn can be related to a similar phenomenon-observed in Gordal fermentation when Ca was added to the brine and may be due to the formation of complexes with flesh components and incrase of consistence, caused by the divalent character of both cations, with the subsequent decrease in difusion (16).

**Table 1:** Overall changes over the period of study in the total sugar content, fructose, and mannitol^1^ according to treatments.

**Treatment**	**Total sugars**		**Fructose**		**Mannitol**	
	**Initial**	**Final**	**Initial**	**Final**	**Initial**	**Final**
Control	0.951 (0.054)^c2^	0.180 (0.004)^a1^	0.427 (0.017)^b2^	0.089 (0.004)^ab1^	0.155 (0.019)^a2^	0.091 (0.001)^a1^
0.050%	0.846 (0.021)^bc2^	0.198 (0.022)^a1^	0.391 (0.019)^ab2^	0.085 (0.003)^ab1^	0.138 (0.010)^a1^	0.113 (0.021)^a1^
0.075%	0.751 (0.055)^ab2^	0.180 (0.008)^a1^	0.390 (0.032)^ab2^	0.077 (0.014)^a1^	0.133 (0.010)^a1^	0.103 (0.008)^a1^
0.100%	0.610 (0.009)^a2^	0.220 (0.006)^a1^	0.350 (0.005)^a2^	0.111 (0.006)^b1^	0.127 (0.014)^a1^	0.109 (0.009)^a1^

1 *Values are averages in g/L, followed by standard error in parenthesis. Sucrose and glucose were omitted from the table since no final residues of them were found at the end of the period of study. Values in the same column followed by different letter superindexes are significantly different at p < 0.05. Values within compounds with different number superindixes for initial and final values are significantly different at p < 0.05*.

**Figure 1:**
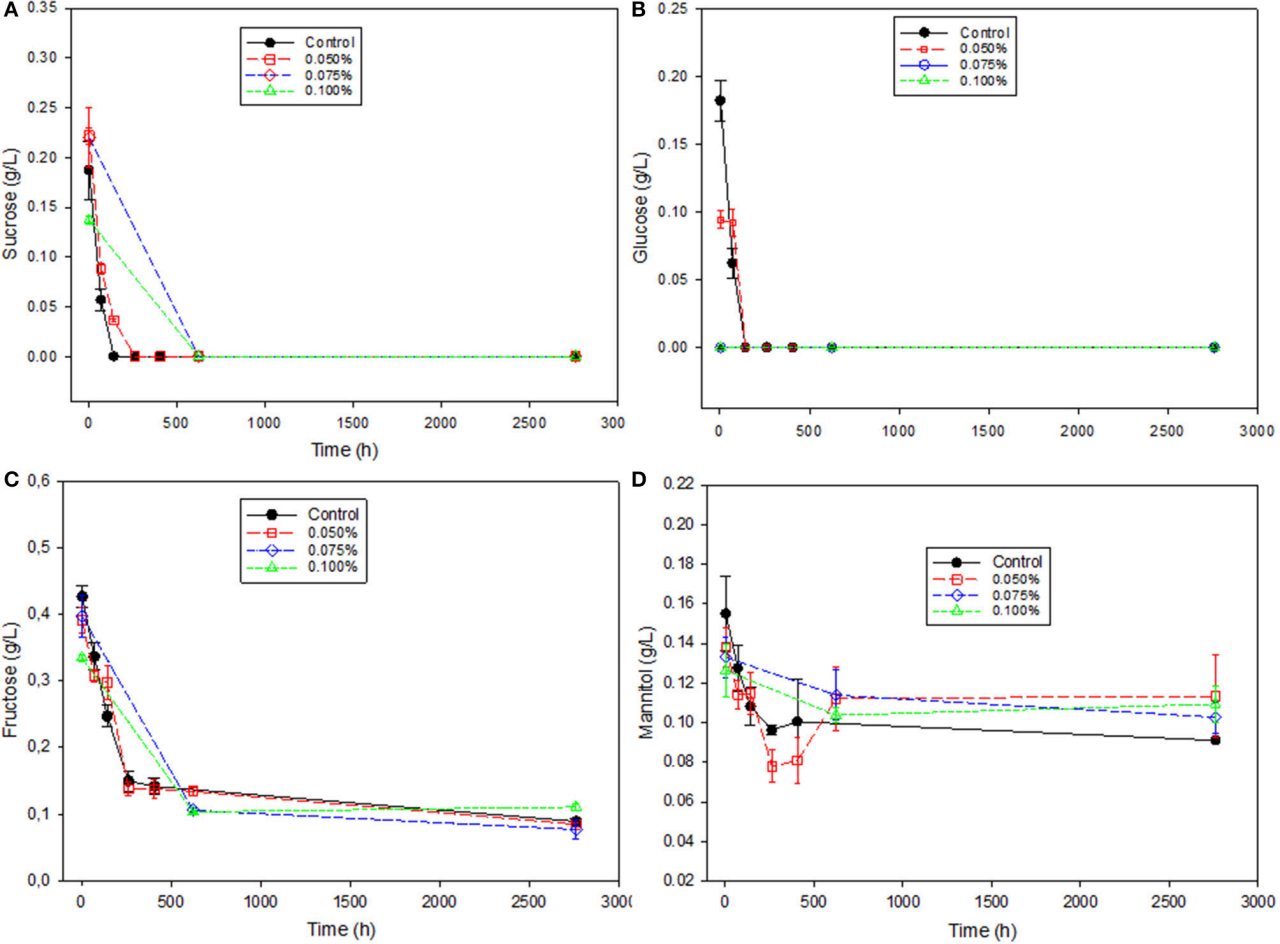
Changes in sugar concentrations in natural black Manzanilla table olive brines over time, according to ZnCl_2_ levels (%, w/v). **(A)** Sucrose; **(B)** Glucose; **(C)** Fructose; and **(D)** Mannitol.

The most abundant contributors to initial total sugars were fructose (0.43 ± 0.02–0.35 ± 0.01 g/L), followed by sucrose (0.18 ± 0.02–0.14 ± 0.00 g/L), glucose (0.11 ± 0.04–not detected g/L), and mannitol (0.16 ± 0.02–0.13 ± 0.01 g/L) (Table 1). The presence of sucrose and glucose was relatively low, and both sugars were entirely consumed soon (Figures 1A,B). Regarding the other sugars, fructose was also used in a high proportion and the final residue, regardless of the treatment, significantly decreased at the end of the study. However, mannitol consumption was slower than any other initially present sugar and was used in lower proportion since the decrease in mannitol during the studied period was significant only in the Control (Table 1 and Figures 1C,D). This indicates that the presence of Zn can hardly improve the manitol consumption. Overall, the total sugar residues at the end of the study was still high but without significant differences among treatments, indicating that the Zn salts were as efficient as the preservatives used by the industry to prevent the sugars' use (except mannitol). In “seasoned” Manzanilla-Aloreña packaging, fructose and mannitol also decreased slowly, with the levels of the last one being unusually high in some treatments (31). The apparent effect of the zinc salt in this work was also in agreement with the results obtained in cracked table olives where the ZnCl_2_ presence led to higher concentrations of residual sugars in brine (due to its selective microbial inhibition) but also to similar trends in their consumptions (10). In general, the residual sugars in the packages represent a source of instability, even in the case of using preservatives and reduce the shelf life of the products (8). The problem is also relevant in the case of cracked Manzanilla-Aloreña table olives, which is an EU DOP, since such instability reduces its commercialization to the area close to their production, particularly when prepared as a fresh-packaged “seasoned” presentation (31).

#### pH, titratable acidity, and organic acids

**Figure 2:**
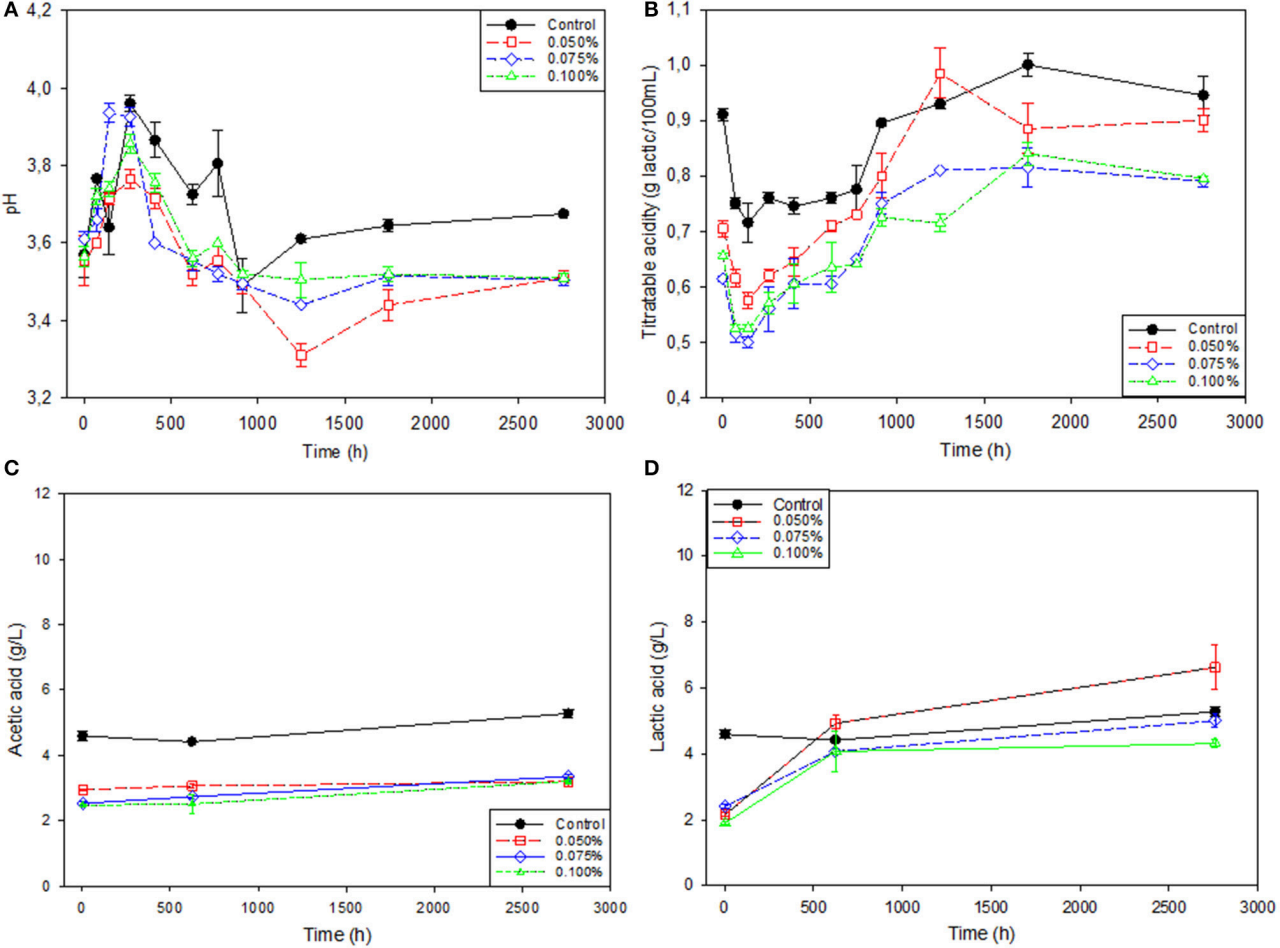
Evolution of pH, titratable acidity and acetic, and lactic acid concentration in natural black Manzanilla table olive brines over time, according to ZnCl_2_ levels (%, w/v). **(A)** pH; **(B)** Titratable acidity; **(C)** Acetic acid; and **(D)** Lactic acid.

The trends followed by these variables were similar in all treatments, although with clear differences among them (Figure 2), with the control showing consistent significantly (*p* < 0.05) higher levels after 1,248 h. Initially, there was a relevant general pH increase (~0.5 units), which reached its maximum at about 250 h after packaging (Figure 2A), simultaneously accompanied by a decrease in titratable acidity, which reached its minimum slightly earlier (Figure 2B). Therefore, the initial changes in pH and titratable acidity were just a matter of equilibrium between the packaging brine and the olive flesh. The phenomenon is normal in olive packaging, where the storage of the final products for 7–15 days before distribution is habitual to allow not only the equilibrium of the physicochemical conditions but also the weight, due to the different brine concentrations used for storage/fermentation and at packaging (8). However, the effect is hardly observable in cracked olives where, due to the fast diffusion of compounds throughout the interior of the olive flesh, the equilibrium is rapid (31). Once equilibrated, there was a relatively fast production of titratable acidity which reached their maxima after ~1,250 h for treatments 0.050 and 0.075% ZnCl_2_ or ~1,750 h for the control and 0.100% ZnCl_2_, with a limited acid formation after these periods. The acidity evolution in the Control was usually significantly (*p* > 0.05) above those in treatments containing Zn (except for 0.050% ZnCl_2_ at 1,248 and 2,760 h), due to its relevant initial sugar content, followed by the treatment containing 0.050% ZnCl_2_. The treatments with higher Zn presence showed similar trends but the lowest (significant at *p* < 0.005) levels at 1,248 h and final titratable acidities (Figure 2B). Therefore, the delay effect on sugar leaching by the progressive presence of Zn in the cover brines was also reflected on a lower titratable acidity. The formation of titratable acidity during shelf life was also observed in previous works and was associated with the presence of LAB whose growth was favored by the reduction of the yeast population by preservatives. However, the use of zinc in Manzanilla-Aloreña enlarged the period of titratable acidity production, possibly due to the high contents of residual nutrients (10). In any case, the excessive LAB presence might not be necessarily favorable since it has also been associated with instability in not lye treated olives (31).

In this work, the titratable acidity was composed of acetic and lactic acids which concentrations were initially most abundant (significant at *p* < 0.05) in the Control than in the treatments containing Zn (Table 2 and Figures 2C,D). The differences in acetic acid between the control and the packages containing Zn scarcely changed during the 4 months due to its similar net increment regardless of treatments (Table 2 and Figure 2C). On the contrary, the lactic acid had a remarkable (significant at *p* < 0.05) initial increment during the first days after brining only in treatments containing Zn (Figure 2D). Later, the production was very slow, except when containing 0.050% ZnCl_2_, which reached the final highest level (significant at *p* < 0.05), followed by the control (Table 2). Therefore, only 0.050% ZnCl_2_ presence stimulated the lactic acid formation.

**Table 2:** Overall changes over the period of study in the acetic and lactic concentrations^1^ according to treatments.

**Treatment**	**Acetic acid**		**Lactic acid**	
	**Initial**	**Final**	**Initial**	**Final**
Control	4.594 (0.125)^c1^	5.276 (0.136)^b2^	4.594 (0.126)^c1^	5.276 (0.136)^a2^
0.050%	2.950 (0.020)^b1^	3.194 (0.135)^a1^	2.156 (0.064)^ab1^	7.125 (0.485)^b2^
0.075%	2.529 (0.044)^a1^	3.352 (0.076)^a2^	2.552 (0.122)^b1^	5.005 (0.199)^a2^
0.100%	2.467 (0.026)^a1^	3.203 (0.050)^a2^	1.708 (0.176)^a1^	4.311 (0.124)^a2^

1 *Values are averages in g/L, followed by standard error in parenthesis. Values in the same column followed by different letter superindexes are significantly different at p < 0.05. Values within compounds with different number superindixes for initial and final values are significantly different at p < 0.05*.

#### Combined acidity, NaCl, and olive firmness

**Figure 3:**
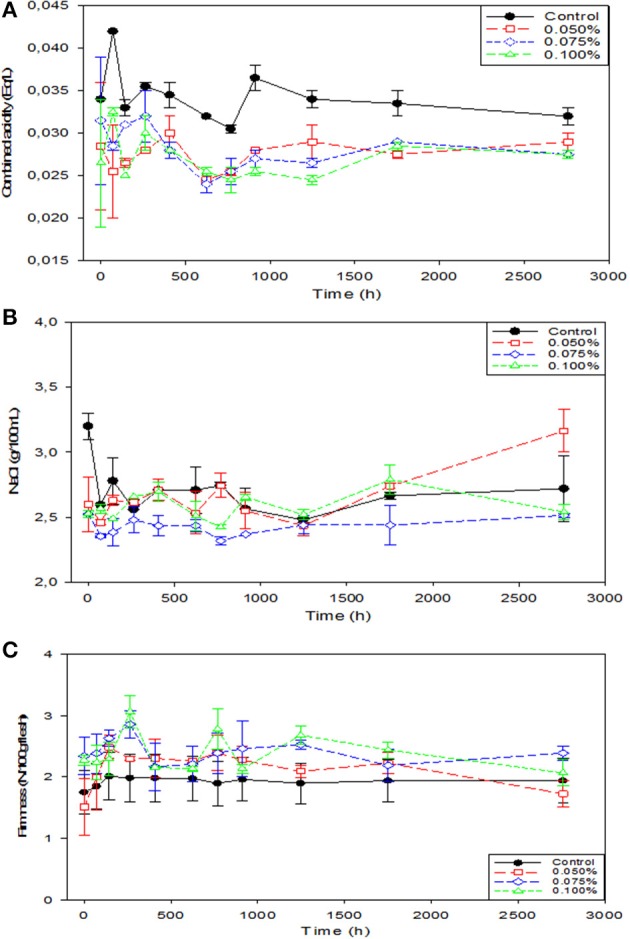
Changes in combined acidity, NaCl concentration and firmness in natural black Manzanilla table olive brines over time, according to ZnCl_2_ levels (%, w/v). **(A)** Combined acidity; **(B)** NaCl; and **(C)** Firmness.

The combined acidity is a measure of the buffer capacity of the brine (8). The highest level was always found in the Control (Figure 3A) with significant (*p* < 0.05) differences at most sampling periods. Hence, the presence of Zn in the brine not only reduced the sugar leaching but also decreased the solubilization of the organic compounds responsible for the combined acidity. In mixtures of chloride salts (KCl, NaCl, and CaCl_2_), the decrease in this parameter was associated with the presence of Ca (also a divalent cation) and its interaction with the olive flesh (32). The Zn may have a different response against olive flesh than Ca, but it can still exert a specific affinity for its components with the retention in the flesh of, at least, a fraction of their hydro soluble compounds. In turn, the reduced combined acidity levels resulted in the lowest pH values (Figure 2A), which can be favorable for the packaged product safety (8).

Concentrations of NaCl, regardless of treatments, followed similar trends (significant difference at *p* < 0.05 only in the initial sampling) and were habitually around 2.5% during the whole period (Figure 3B). This stability is reasonable due to the inorganic nature of salt. Panagou et al. (33) and Panagou (34) did not find either any significant change of this parameter in dry-salted olives of the Thassos variety when the fruits were stored under modified atmospheres at 4 and 20°C.

The firmness of the treatments containing Zn was mainly above that of the Control and in some cases significantly (*p* < 0.05) higher (Figure 3C), although at the end of the period this had an intermediated value. Therefore, the Zn, as a divalent cation, in addition to preventing the organic matter solubilization, formed stable complexes with the wall structure of the olive cells and increased the product hardness (~0.5 kN/100 g olive flesh), at least during most of the packaging period. Interestingly, firmness of control scarcely changed with time despite the acid medium. This trend is in contrast with the progressive degradation of firmness in seasoned olives, reported by Fernández-Bolaños et al. (35). In this case, the effect was attributed to both the enzymes coming from the constituents of the dressing products (garlic, lemon, etc.) and those from the endogenous olive flesh since the influence was noticed in either the presence or absence of seasoning materials. The authors related the changes in texture to the degradation of polysaccharides, caused by α-cellulase and polygalacturonase present in the flesh which were not so active as in the case of natural black Manzanilla olives.

#### Color

**Figure 4:**
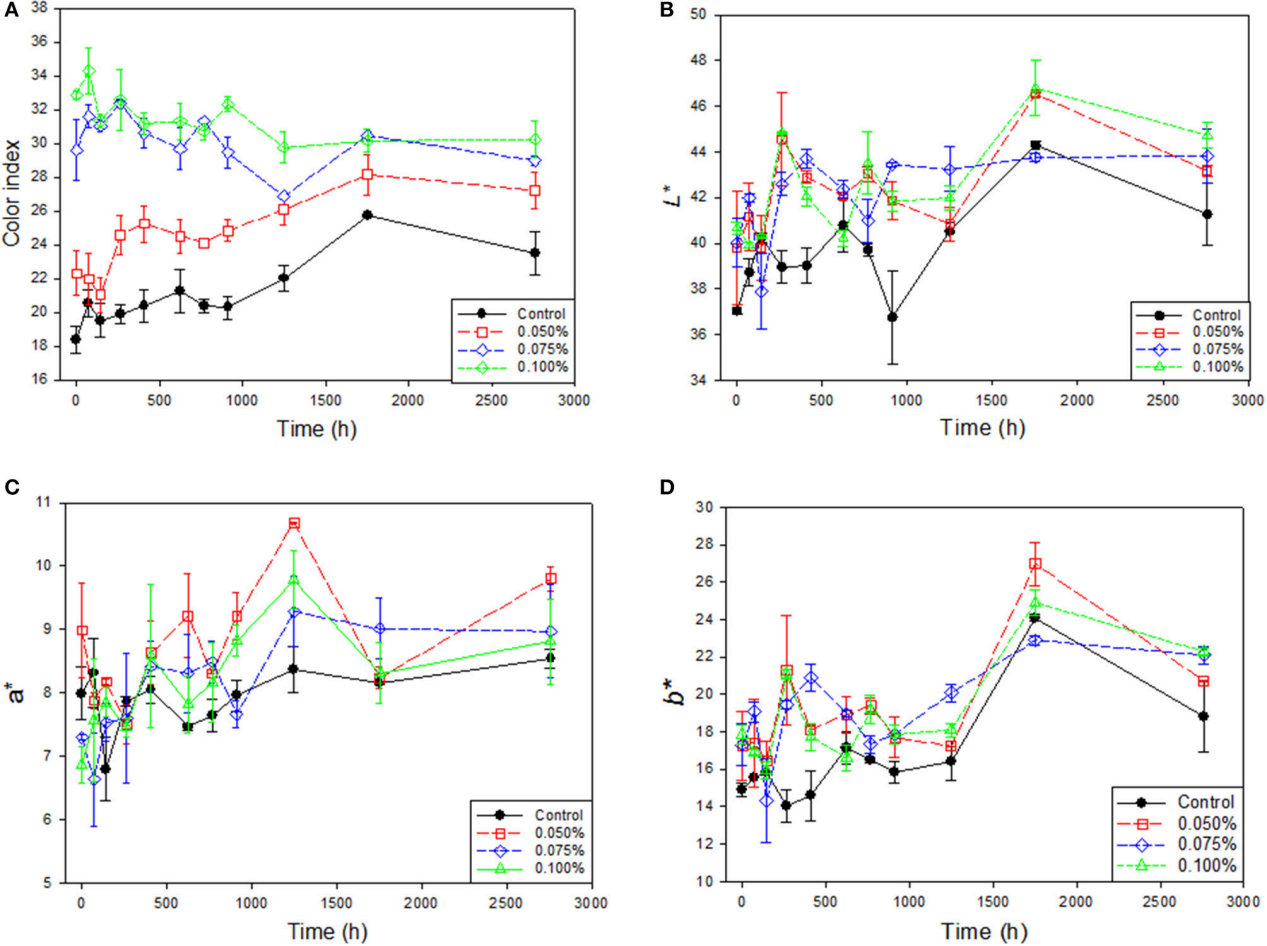
Evolution of color index, L*, a*, and b* (unweighted means) in natural black Manzanilla table olive brines over time, according to ZnCl_2_ concentrations (%, w/v). **(A)** Color index; **(B)** L*; **(C)** a*; and **(D)** b*.

The changes in color were followed by studying color index and the CIE L^*^, a^*^, b^*^. Initially, the effect of zinc was outstanding on the color index which was rather high (significant at *p* < 0.05) and similar in samples containing 0.075 and 0.100% ZnCl_2_; later, slightly decreased and stabilized. On the contrary, the color index in the Control and 0.050% ZnCl_2_ increased over time up to ≈1750 h. After this time, they became stable and followed a similar trend (without significant individual changes) than the other two Zn treatments (Figure 4A). The gradation of the color index, in ascendant value order, was: Control, 0.050%, 0.075–0.100% ZnCl_2_, with significant (*p* < 0.05) differences over most of the studied period. Therefore, in general, Zn presence led to higher color indexes.

The influence of ZnCl_2_ was also observed on the CIE^*^ parameters which followed similar trends with the control always below (in some cases significantly at *p* < 0.05) the treatments with Zn (Figures 4B–D), and final values above those showed at packaging but without significant differences. There was a period (between 250 and 500 h) where, apparently, the Zn effect was more intense (significant at *p* < 0.05) on L^*^ and b^*^ which values, on the contrary, always decreased at the end of the period. Changes in a^*^ followed a similar trend, although the average values of Control were among the lowest during most of the studied period. The effect on both L^*^ and b^*^ paramenters should be attributed to the presence of Zn since the levels pH (high) and acidity (low) would have influenced on opposed direction. According to Arroyo-López et al. (31), in cracked Manzanilla-Aloreña, the only parameter affected was a^*^ which had a marked increase due to surface color change from green to more or less intense yellow. This transformation was caused by the degradation of chlorophylls and carotenoids into colorless or brownish compounds (36). In this work, the main visual change in the natural black Manzanilla olive color was from purple to pink due to the degradation of anthocyanins.

### Changes in the microbiological populations

**Figure 5:**
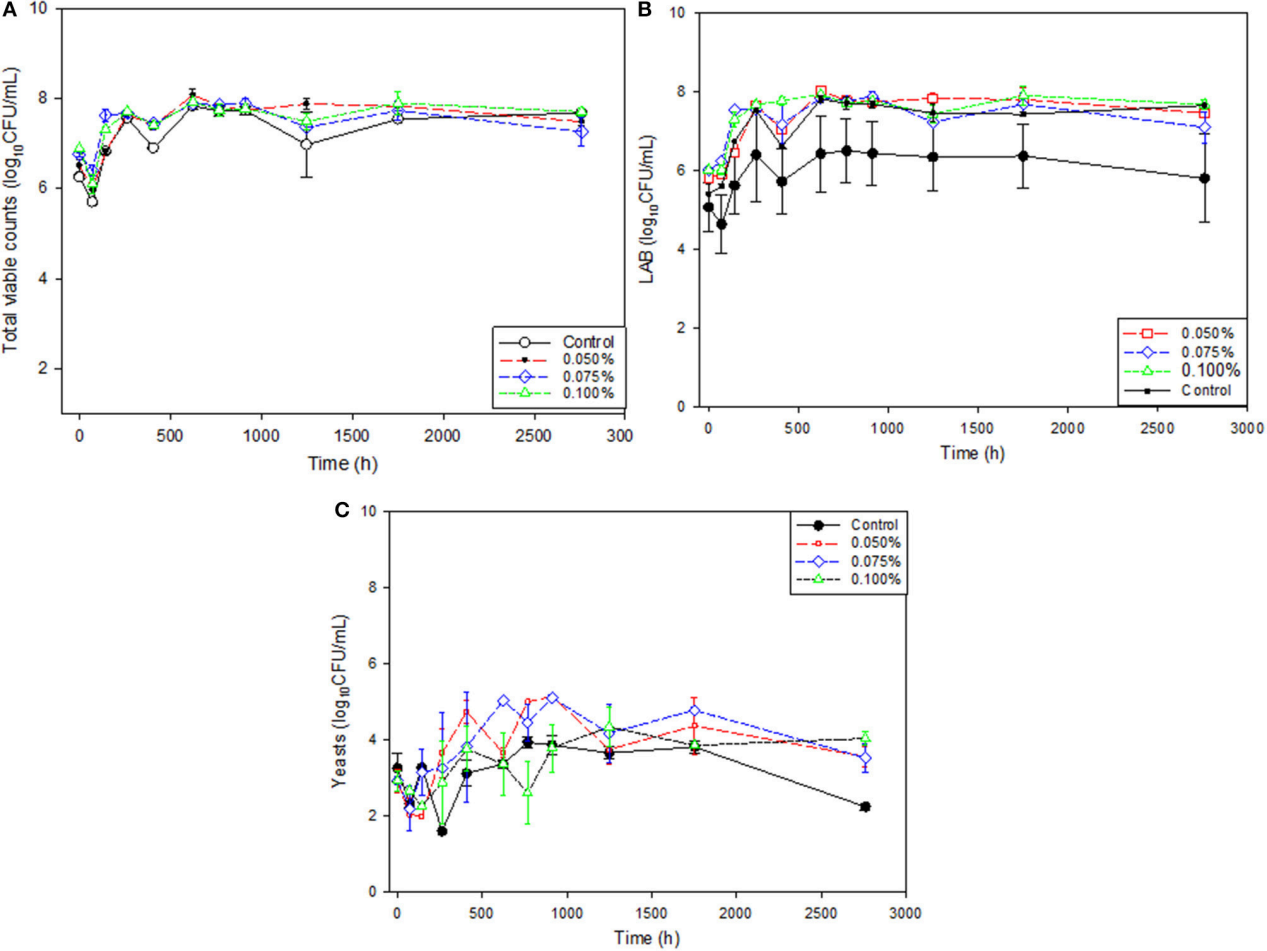
Changes in microbial populations (unweighted means) in natural black Manzanilla table olive brines over time, according to ZnCl_2_ concentrations (%, w/v). **(A)** total counts; **(B)** LABs; and **(C)** Yeasts.

The study of the packaged olive microbiota was based on total viable counts, LAB, and yeasts (Figure 5) since no *Enterobacteriaceae* were found. Total viable counts had an initial decrease, but the population grew soon to reach a level close to 8 log_10_CFU/mL which remained practically stable throughout the 4 months, independently of treatment (Figure 5A). To notice that the effect of zinc on total viable counts was similar to that observed for preservatives (sorbate + benzoate) in the Control. This microbial population was mainly composed of LAB and yeasts. LAB population was remarkable high (~5–6 log_10_CFU/mL) from the beginning and increased after a short period to reach 7.5–8 log_10_CFU/mL, in treatments containing Zn, but only around 7 log_10_CFU/mL, in the Control. Later, their counts remained stable during the rest of the period, although maintaining the differences (Figure 5B). Therefore, the LAB trends were quite similar, but the average populations in the Control were always below those treatments containing Zn, although the differences were mostly non-significant due to the high variability between replicate. The presence of the metal stimulated the LAB growth slightly but did not encourage simultaneously the titratable acidity production since its average levels were below those in the Control throughout the period, except in one sampling point (Figure 2B). Molecular analysis at the end of the period revealed that LAB population was mainly composed of *L. pentosus* (39%) and *L. plantarum* (61%), but their distribution was not related to the ZnCl_2_ contents.

The yeast population in the Control was always similar or only slightly below than in those treatments containing zinc (except in one sampling point), without any systematic difference based on the Zn concentration (Figure 5C). Their populations had a small initial decrease but soon recovered and stabilized around 3–4 log_10_CFU/mL with a slight decrease in the last counting. Therefore, the presence of ZnCl_2_ had an approximately inhibitory similar effect on yeast than the preservatives.

### Sensory analysis and nutritional implications

**Figure 6:**
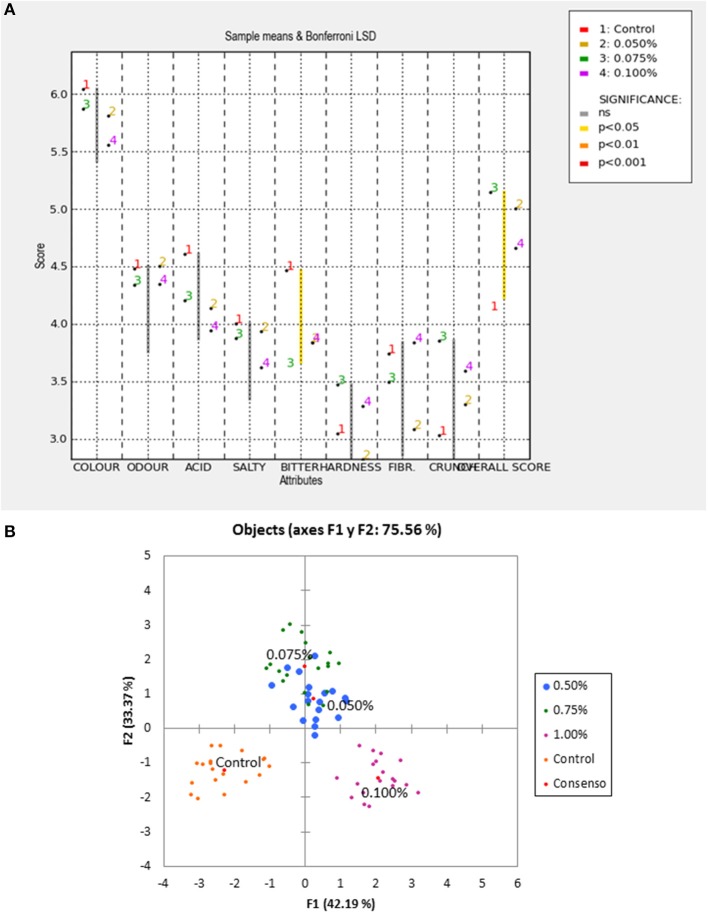
Sensory analysis of natural black Manzanilla table olive, according to ZnCl_2_ concentrations (%, w/v) in the packaging brines **(A)** significant descriptors and **(B)** segregation of treatments by panelists.

The results of the sensory analysis by the panel were subjected to both panel performance and characterization of products. In a first step, the data for each attribute were subjected to an ANOVA considering the effects of treatments as fixed and those of assessors, sessions and their interaction as random. Only bitter and overall scores in the Control and 0.075% ZnCl_2_ treatment resulted significantly different at *p* ≤ 0.05 (Figure 6A). A procrustean analysis of the scores, using XLSTAT, showed that, in general, the panelists segregated the treatments correctly (Figure 6B). However, the interest was mainly focused on the characterization of the products according to the descriptors used in the sensory evaluation. Such a task was accomplished by observing the adjusted mean values from the corresponding ANOVA test (Table 3). The highest overall score (in blue) was given to the product containing 0.075% ZnCl_2_, the lowest corresponded to control (in red), while the other treatments had intermediate scores. The highest scores for acid and bitter were obtained for the Control. On the contrary, the lowest values for fibrousness were found in treatment with only 0.050% ZnCl_2_.

**Table 3:** Average scores of descriptors adjusted for products after ANOVA application to the original data.

	**Crunch**	**Overall score**	**Hardness**	**FIBR**	**Color**	**Salty**	**Odor**	**Acid**	**Bitter**
0.100%	3.600	4.663	3.289	3.846	5.563	3.626	4.351	3.949	3.840
0.075%	3.861	5.146	3.475	3.496	5.874	3.881	4.340	4.205	3.625
0.050%	3.307	5.009	2.826	3.086	5.814	3.940	4.505	4.142	3.844
Control	3.040	4.118	3.054	3.746	6.046	4.004	4.486	4.611	4.470

*CRUNCH, crunchiness; FIBR, fibrousness. The highest (in blue) and the lowest (in red) overall score*.

**Figure 7:**
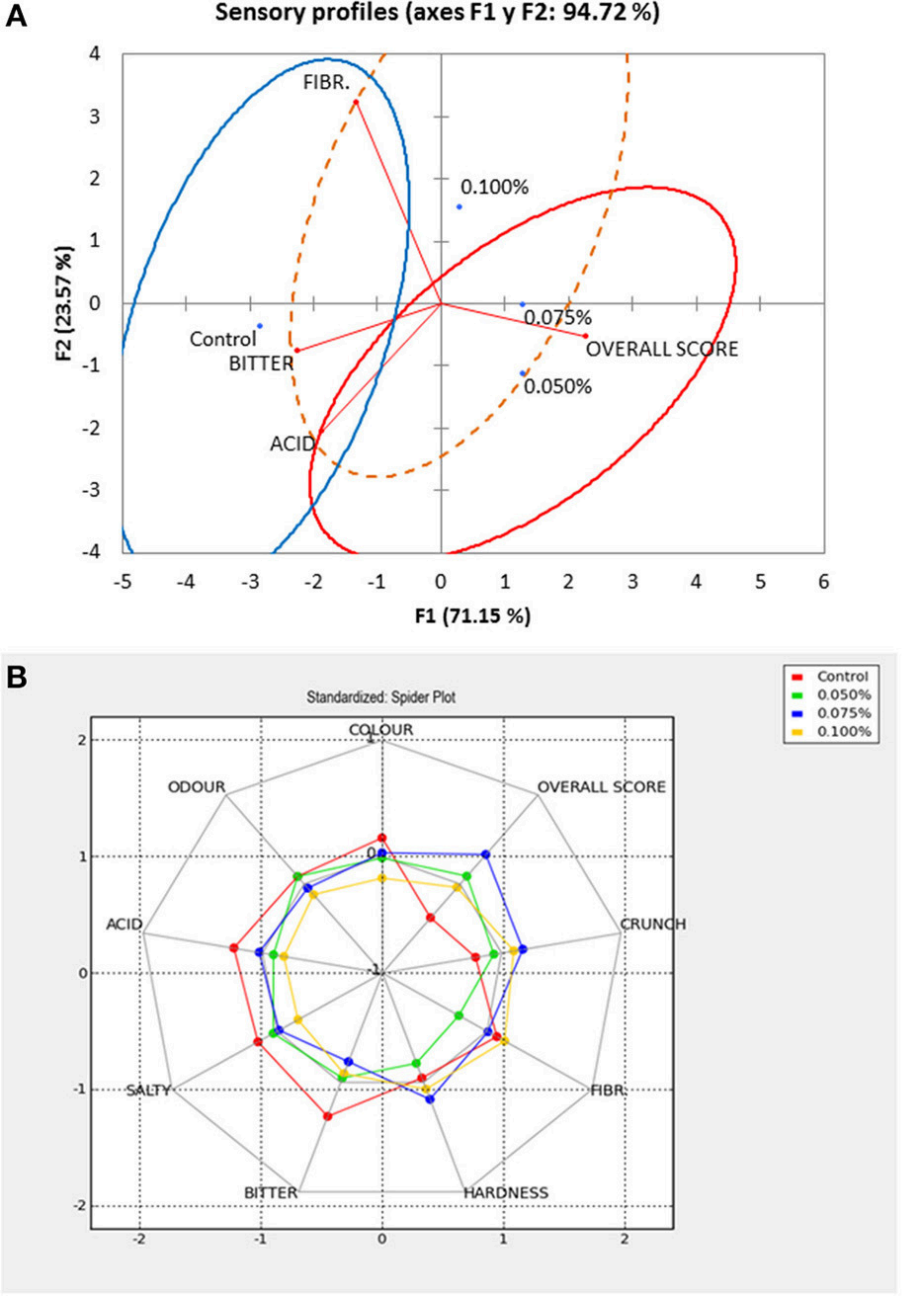
Sensory analysis of natural black Manzanilla table olive, according to ZnCl_2_ concentrations (%, w/v) in the packaging brines **(A)** Projection of the different treatments scores on the first two Principal Components (sensory profiles) and their ellipse confident limits (*p* = 0.05). **(B)** Spider plot, based on all descriptors, to characterize the diverse products, using standardized average scores.

The contributions of descriptors to discriminate among treatments agreed with the previous observations (Figure 6A) and indicated that only overall score and bitter had significant discriminant powers. However, for more extensive product characterization, fibrousness and acid should also be considered (although they had a lower significance, *p* ≤ 0.100) since they had relevant low and high scores, respectively. Applying PCA, a projection of the loadings of the attributes (filtered for only those significant at *p* ≤ 0.100) and the treatment scores on the two first components, three groups and their 95% confidence limits were obtained (Figure 7A). The first corresponded to the control and was mainly positively associated to bitter as well as to acid and negatively to the overall score. The second group consisted of treatments using 0.050 and 0.075% ZnCl_2_ which were linked to the highest overall scores and the lowest bitter and fibrousness levels. Finally, treatment with 0.100% ZnCl_2_, was characterized by low acid and bitter scores, intermediate fibrousness and overall scores; however, it was not well segregated from the other groups with which overlapped extensively. A characterization of the diverse products based on all descriptors, using standardized average scores, was also obtained by the spider plot (Figure 7B). The control had the highest average scores in color, odor, acid, salty, bitter (mainly), and fibrousness.

On the contrary, the treatment containing 0.075% ZnCl_2_ had the highest hardness, crunchiness and overall scores. Therefore, the sensory analysis showed that the presence of 0.075% ZnCl_2_ markedly improved the sensory quality of the product. Notably, the addition of the Zn contributed to the reduction of the bitter taste which was a sensation strongly and negatively related to overall score (Figure 7A).

The treatments were also pairwise compared, applying a methodology similar to that used by the h-index estimation. The test consisted of two essays: the first was performed with the aim of knowing the discriminant power of the assessors while the second had the objective of analyzing their preferences. In the first case, the control was considered different from treatments containing 0.100, 0.075, and 0.050% ZnCl_2_ by 95, 84, and 95% of the assessors; therefore, the products containing Zn were differentiated from the control (with preservatives). When the comparison was made between presentations containing Zn, treatment with 0.050% ZnCl_2_ was considered different from 0.075 to 0.100% by 79 and 95% of the panelists, respectively. The difference between 0.075 and 0.100% was also perceived by 84% of the assessors. Therefore, it may be concluded that the treatments containing Zn are different from control and among them.

Concerning preferences, when the treatments containing progressive lower contents were compared to the Control, 53, 79, and 58% of the assessors preferred those containing 0.050, 0.075, and 0.100% ZnCl_2_, respectively. The significance of the responses, evaluated by a χ^2^ test (χ^2^ = 0.277, *p* = 0.599; χ^2^ = 33.524, *p* < 0.0001; and χ^2^ = 6.927, *p* = 0.122, respectively), showed that only the treatment containing 0.075% ZnCl_2_ was significantly preferred against the Control. Therefore, in general, the treatments added with Zn were differentiated from the control, but only the treatment added with 0.075% ZnCl_2_ was statistically preferred over it. Treatments containing ZnCl_2_ were also compared among them. The treatment containing 0.075% was significantly preferred against that with 0.100% by 68% (χ^2^ = 13.572, *p* = 0.0002) but only by 47% vs. that with 0.050% (χ^2^ = 0.277, *p* = 0.599). However, there was no statistical difference between 0.050 and 0.100% treatments. The comparison among Zn treatments was not as clear as that against the control but showed that olives containing 0.075% ZnCl_2_ were significantly preferred against those with 0.100% but not against 0.050% ZnCl_2_. The results obtained in this work agree with those previously observed in “seasoned” *Aloreña de Málaga* packaged in brines added with Zn in which the packages containing 0.075% ZnCl_2_ were also preferred to any other treatment. Furthermore, in the experiment with *Aloreña de Málaga*, the highest scores in the absence of Zn were also given to bitter, but the values decreased as the element proportion in treatments increased (10).

Also working with Manzanilla *Aloreña de Málaga*, the correlations between bitter scores and Zn contents in flesh were negative. Besides, when the sensory data were subjected to PLS analysis, the projections of attribute loadings and treatments scores on the first two PCs positively related the control to bitter and the kinesthetic sensations while the product with 0.075% ZnCl_2_ was associated with high titratable acidity and acid scores (11). In a first approach for the preparation of green Spanish-style olive packaging containing ZnCl_2_, however, no effect of Zn content was observed, possibly due to the lye treatments used for debittering (12). Further comparisons between packaged green olives using ZnCl_2_ and potassium sorbate or sodium benzoate showed that the panelists preferred the olives packaged with Zn against those containing the classical preservatives but no relationship with bitter was found (private communication). Therefore, regarding sensory characteristics, the presence of ZnCl_2_ has demonstrated a clear mitigating effect on the bitter perception mainly in directly brined olives, in which the residual sensation of this attribute is still notable.

The inclusion of this important nutritional element in such presentations could lead to new and healthier table olive presentations. The product will increase the Zn intake in ~8–20 mg Zn/100 g olive flesh (considering its equilibrium between brine and flesh moisture). In the case of the most appropriate concentration (0.075% ZnCl_2_ in cover brine), the content would be ~15 mg/100 g olive flesh which, for a reasonable service of about 7 olives, will represent a contribution of about half the current recommended Zn daily intake for an adult (10 mg/day) (37). However, polyphenols are among the compounds that decrease the Zn absorption (38). As the concentrations of polyphenols are particularly high in directly brined natural black olives (7), a study of the bioavailability of Zn in this product would be convenient before a more accurate estimation of the potential nutritional improvement of natural black olive zinc fortified presentations could be established.

## Conclusions

This work has developed a novel Zn fortified natural black Manzanilla olive preparation. The presence of Zn decreased sugar diffusion from olives, had a comparable effect on yeasts than preservatives, stimulated LAB growth slightly and lactic acid production (at moderate Zn concentration), led to high color index, and improved sensory characteristics of the product considerably. Although other factors like pH, acidity or microbial growth might also have been influenced the above-mentioned parameters, their changes were, in turn, related to the presence of Zn and, therefore, indirectly associated to the presence of Zn. Treatments with zinc were differentiated and preferred against the control since the mineral element reduced the bitter perception of the packaged olives. Considering that bitter is one of the primary negative table olive attributes for consumers, the addition of a moderate proportion of ZnCl_2_ to these products will improve sensibly not only their healthy characteristics but also their appreciation and, as a result, contribute to the expansion of the natural table olive market.

## Author contributions

JB-G, FR-G, VR-G, and AB-C performed the experiments, participated in the acquisition, analysis and interpretation of the data, and approved the final version of the paper. JB-G, FA-L, and AG-F supervised the laboratory work, participated in the analysis and interpretation of the data, drafted the manuscript, and approved the final version of the paper.

### Conflict of interest statement

The authors declare that the research was conducted in the absence of any commercial or financial relationships that could be construed as a potential conflict of interest.
